# Interval follow up of a 4-day pilot program to implement the WHO surgical safety checklist at a Congolese hospital

**DOI:** 10.1186/s12992-017-0266-0

**Published:** 2017-06-29

**Authors:** Michelle C. White, Jennifer Peterschmidt, James Callahan, J. Edward Fitzgerald, Kristin L. Close

**Affiliations:** 1 Mercy Ships, Department of Medical Capacity Building, Port of Pointe Noire, Democratic Republic of Congo; 2 Mercy Ships, Department of Medical Capacity Building, Port of Cotonou, Benin; 3 Lifebox Foundation, London, UK

**Keywords:** Global surgery, Patient safety, Education, Checklist

## Abstract

**Background:**

The World Health Organisation Surgical Safety Checklist (SSC) improves surgical outcomes and the research question is no longer ‘does the SSC work?’ but, ‘how to make the SSC work?’ Evidence for implementation strategies in low-income countries is sparse and existing strategies are heavily based on long-term external support. Short but effective implementation programs are required if widespread scale up is to be achieved. We designed and delivered a four-day pilot SSC training course at a single hospital centre in the Republic of Congo, and evaluated the implementation after one year. We hypothesised that participants would still be using the checklist over 50% of the time.

**Method:**

We taught the four-day SSC training course at Dolisie hospital in February 2014, and undertook a mixed methods impact evaluation based on the Kirkpatrick model in May 2015. SSC implementation was evaluated using self-reported questionnaire with a 3 point Likert scale to assess six key process measures. Learning, behaviour, organisational change and facilitators and inhibitors to change were evaluated with questionnaires, interviews and focus group discussion.

**Results:**

Seventeen individuals participated in the training and seven (40%) were available for impact evaluation at 15 months. No participant had used the SSC prior to training. Over half the participants were following the six processes measures always or most of the time: confirmation of patient identity and the surgical procedure (57%), assessment of difficult intubation risk (72%), assessment of the risk of major blood loss (86%), antibiotic prophylaxis given before skin incision (86%), use of a pulse oximeter (86%), and counting sponges and instruments (71%). All participants reported positive improvements in teamwork, organisation and safe anesthesia. Most participants reported they worked in helpful, supportive and respectful atmosphere; and could speak up if they saw something that might harm a patient. However, less than half felt able to challenge those in authority.

**Conclusion:**

Our study demonstrates that a 4-day pilot course for SSC implementation resulted in over 50% of participants using the SSC at 15 months, positive changes in learning, behaviour and organisational change, but less impact on hierarchical culture. The next step is to test our novel implementation strategy in a larger hospital setting.

## Background

The World Health Organisation (WHO) Surgical Safety Checklist (SSC) improves compliance with basic safety processes and surgical outcomes [1, 2] but the most effective methods of implementation in low and middle-income countries (LMIC) are unknown. Reports of successful SSC implementation in LMICs exist [3–7], but rely on significant time and resource commitments from high-income country (HIC) providers, which limits wide-spread implementation. Therefore, with scale-up in mind, we piloted a four-day SSC training course using a small team of HIC providers.

Team training and supportive hospital leadership are important for sustained implementation [8]. Barriers to successful SSC implementation include lack of adaptation to local practice, paucity of buy-in due to poor understanding, general lack of supplies and functioning equipment [9, 10]. Therefore our four-day SSC training was based on the principles of (a) understanding the rationale for the SSC; (b) local ownership through locally-driven adaptation; (c) donation of equipment; (d) team training and operating room simulation. We aimed to pilot the four-day course at the main hospital in Dolisie, and hypothesised that one year after the course, over 50% of participants would still be using the SSC and following six basic safety processes as described by Haynes et al. [1]; and that there would be sustained positive changes in learning, behaviour and organisational practice.

## Methods

The Minister of Health of the Republic of Congo gave permission for the training program and the subsequent evaluation as part of a Mercy Ships countrywide healthcare education program. Individuals gave verbal consent to participate but written signed consent was not required. The study was given ethics approval by the Mercy Ships Institutional Review Board (study number MS2016004) and the requirement for individual written consent was waived.

### Baseline assessment

Mercy Ships is a global charity operating the world’s largest non-governmental hospital ship, the *Africa Mercy*. Mercy Ships visits coastal African countries at the invitation of the President and works closely with the Ministry of Health to deliver surgical services, and training. From August 2013 to June 2014 Mercy Ships was docked in Pointe Noire, Republic of Congo. The Mercy Ships Project Manager undertook a one-day baseline assessment visit, four months prior to the training.

### Participants and setting

The Republic of Congo has a population of 4.6 million [11], and Dolisie is the third largest city. The hospital in Dolisie serves a population of approximately 90,000 people. At the time of the baseline assessment there was one surgeon, two obstetricians and five nurse anaesthetists. No one had ever heard of the SSC, there were no pulse oximeters available for three operating rooms and the recovery room, and nurses did not know how to count needles, sponges and instruments. There was no formal process for discussing the risk or difficult intubation or estimated blood loss. The SSC training was explained to the hospital director, classroom facilities identified and dates agreed for training.

### Training programme

**Fig. 1:**
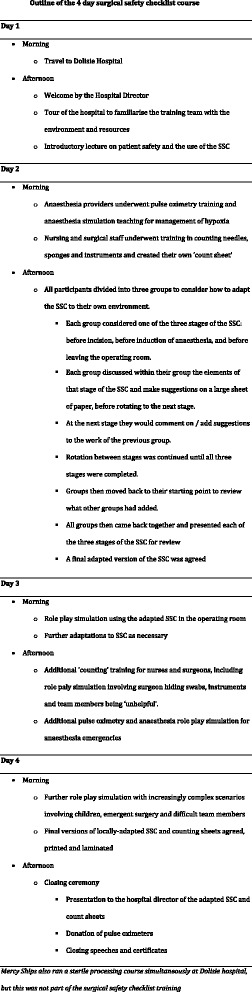
Outline of the 4 day surgical safety checklist course

The SSC course outline is given in Fig. 1. The training occurred over four days in February 2014, and used five training facilitators from HICs. Three were Mercy Ships crew with experience teaching the SSC (a physician anaesthetist, operating room nurse and training project manager); one British trainee surgeon, and one French teacher providing support and translation.

Participants were all operating team personnel at Dolisie hospital, and were invited to attend the training by the hospital director.

The Mercy Ships project manager and physician anaesthetist returned to Dolisie hospital three months after training to encourage continued use of the SSC and trouble shoot obstacles to implementation. Data was not formally collected as part of this visit which was primarily for training purposes but field notes were made in notebooks.

### Impact evaluation

We used a mixed-methods design to assess impact of SSC implementation at 15 months based on the Kirkpatrick model for evaluation [12], (Table 1). Structured interviews, focused group discussion and questionnaires were used to collect data.

**Table 1:** Kirkpatrick model for evaluating effects of training courses

Level 1: Reaction	Participants perception of the course (enjoyment, relevance and engagement)
Level 2: Learning	Acquired knowledge, skills, attitude, confidence, commitment
Level 3: Behaviour	Translation of knowledge and skills into routine personal practice
Level 4: Results	The ultimate goal; organisational change and improved patient outcome

All participants still working at the hospital 15 months after training were asked by the hospital director to attend the impact evaluation. Interviews, focused group discussion, and observations in the operating room took place in the hospital and were conducted by the authors KC and MW. MW is a physician anaesthetist and KC is the training projects manager. Both KC and MW participated in the original SSC training. Participants were interviewed once in French with translation where necessary, and interviews lasted 15–30 min. There was one focused group discussion. Responses were not recorded and transcribed due to budget constraints but instead recorded with pen and paper contemporaneously.

Structured interviews and focused group discussion were based around the following questions:

1. What were the most important things you learnt from the surgical safety checklist training?
2. Have you made any changes in your personal practice since the training?
3. Have you noticed any changes in your hospital since the training?
4. Did anything help or hinder the changes that were made?

Questions 1, 2 and 3 correspond to Kirkpatrick level 2,3 and 4 evaluation respectively. Question 4 aimed to identify facilitators and inhibitors to change to aid future course development.

**Fig. 2:**
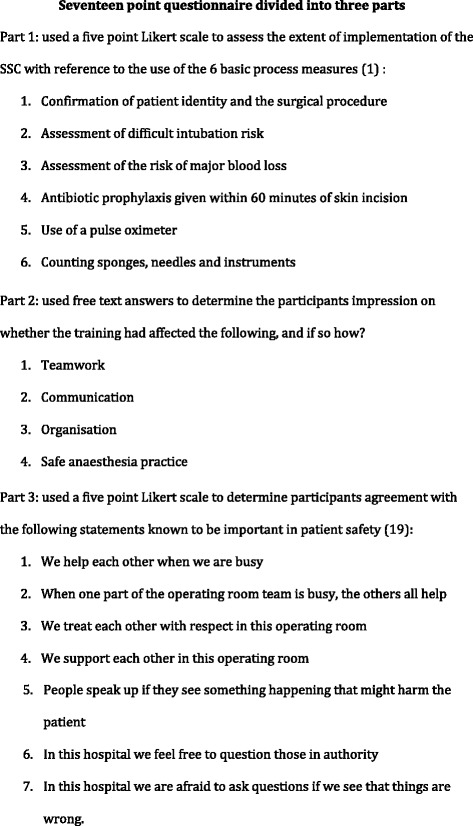
Seventeen point questionnaire divided into three parts

Participants also completed an anonymous questionnaire, written in French, made up of 17 questions in three parts (see Figure 2). Part 1 was designed to evaluate extent of SSC implementation, and parts 2 and 3 to further determine learning, behaviour and organisational change (Kirkpatrick Level 2,3 and 4 changes).

Observations in the operating room were planned to visually assess SCC implementation and recorded in field notebooks.

### Hypothesis and primary outcome measure

The primary hypothesis was that after one year, over 50% of participants would be following the six basic safety processes [1] most of the time. Therefore the primary outcome measure was the number of participants following each safety measure, ‘always’ or ‘most of the time’.

The secondary hypothesis was that the SSC would lead to positive changes in learning, behaviour and organisational change (Kirkpatrick level 2, 3, and 4) evaluation.

### Statistical analysis

To test the primary hypothesis, simple descriptive statistics were used. To test the secondary hypothesis we use mixed methods: quantitative data was analysed with descriptive statistics; and qualitative data was manually themed the interviews, focus group discussion, and questionnaire responses for analysis and reporting of descriptive patterns [13].

## Results

Between the time of the baseline assessment in October 2013 to the SSC training in February 2014, the general surgeon and one obstetrician had left the hospital and there was an increase from five to seven nurse anaesthetists. Therefore 17 individuals (all the operating room staff) participated in the initial SSC training in February 2014 (1 obstetrician, 7 nurse anaesthetists and 9 operating room nurses, see Table 2).

**Table 2:** Numbers of participants in surgical safety checklist training and number followed-up for impact evaluation at 15 months

	Number who participated in training	Number followed-up at 15 months	Reason for lack of follow-up
Obstetrician	1	0	Had left and not been replaced
Anaesthesia nurses	7	3	1 was working on the admissions unit and unable to attend the interview; 2 were transferring a patient to a hospital 4 h away; 1 was on vacation
Operating room nurses	9	4	1 had left and not been replaced; 4 were unaccounted for and colleagues were unwilling to say where they were

At the 3 month evaluation the SSC was seen on the wall of the operating room and staff (Hospital Director, nurse anaesthetists and operating room nurses) reported using the SSC including pulse oximetry and counting, and showed evidence of this in practice. A large pile of completed ‘counting sheets’ was seen.

At the impact evaluation in June 2015 only 7/17 (40%) participants were available for interview, 3 nurse anaesthetists and 4 operating room nurses. The obstetrician had left and had been replaced by temporary surgeons / obstetricians who came on a rotational basis to provide surgical cover. Details of training participants are given in Table 2.

At 15 months after training, the six basic safety processes were being performed by more than half of the attending participants either always or most of the time: confirmation of patient identity and surgical procedure (57% of participants), assessment of difficult intubation risk (72% of participants), assessment of the risk of major blood loss (86% of participants), antibiotic prophylaxis given before skin incision (86% of participants), use of a pulse oximeter (86% of participants), and counting (71% of participants). Details are given in Table 3.

**Table 3:** Frequency of self reported use of the 6 basic safety process measures 15 months after training. Values are given as numbers (percentage)

	Always	Most of the time	Sometimes	Occasionally	Never	No response
1. Is the identity of the patient verified with the surgical team before starting surgery	4 (57%)	0	2 (29%)	1 (14%)	0	0
2. Is the risk of difficult intubation evaluated before surgery?	3 (43%)	2 (29%)	1 (14%)	0	0	1 (14%)
3. Is the risk of large blood loss evaluated before surgery?	2 (29%)	4 (57%)	1 (14%)	0	0	0
4. Is a pulse oximeter used in the OR?	6 (86%)	0	1 (14%)	0	0	0
5. Are prophylactic antibiotics given before surgery?	3 (43%)	3 (43%)	1 (14%)	0	0	0
6a. Are needles, sponges and instruments counted before and after surgery?	5 (71%)	0	1 (14%)	1 (14%)	0	0

### Kirkpatrick levels 2,3 and 4 results from interviews and focussed group discussion responses

The most important things learnt recalled by participants were, introducing themselves to each other, confirming the patient identity and site of surgery, and counting (Kirkpatrick level 2). This was backed up by responses to the questions regarding personal and organisational change (Kirkpatrick level 3 and 4).

Several participants reported that when they introduced themselves in front of the patient, and verified the patient’s identity and operation site, then the patient was reassured and visibly calmed down. Participants also reported that visiting surgeons were asked to introduce themselves and participate in this process in front of the patient. Counting and giving antibiotics on time were also reported as personal and organisational changes. Further details are given in Table 4.

**Table 4:** Summary of the five most common themed responses to Kirkpatrick level 2, 3 and 4 questions

1. What were the most important things you learnt from the surgical safety checklist training? (Kirkpatrick level 2)	• Introducing ourselves • Checking identity of patient and surgery planned • Counting • Teamwork and sharing knowledge • Using the pulse oximeter
2. Have you made any changes in your personal practice since the training? (Kirkpatrick level 3)	• Counting • Checking identity of patient and surgery planned • Introducing ourselves • Giving antibiotics pre-skin incision instead of afterwards • Welcoming the patient and reassuring them
3. Have you noticed any changes in your hospital since the training? (Kirkpatrick level 4)	• We now accompany the patient from the ward to the operating room • Introducing ourselves in front of the patient which calms them down and reassures them • Demanding that visiting surgeons introduce themselves • Giving antibiotics before skin incision instead of at the end of the surgery • Counting

### Kirkpatrick level 3 and 4 results from the questionnaire responses

#### Questionnaire part 2 responses

A selection of the commonest participants free text responses to questions asking about the impact of SSC training on perceptions of teamwork, communication, organisation and safe anaesthesia are given in Table 5. All seven participants reported the training had a positive effect on teamwork, organisation and safe anaesthesia practices. Six out of seven reported a positive effect on communication; and one did not answer the question.

**Table 5:** Free text responses to questions asking if the training had impacted participant’s perception of teamwork, communication and safe anaesthesia in the operating room

Teamwork	• We are now all on the same page • We can help each other and encourage each other • We now have a team atmosphere, suggestions and mutual exchanges • Everybody in the operating knows their role
Communication	• We are now free to disagree • Before I would just get angry if people weren’t interested, now we can talk about it • Professional information is now shared • We are nicer to each other
Organisation	• It has brought our team to life • We are more precise • More efficient
Safe anaesthesia	• It is safer now because before, the anaesthetist didn’t tell us if there was problems • We always ask now if anaesthesia is ready and if anything is missing • Because of the pulse oximeter I feel safer giving anaesthesia • I think there are less deaths now

#### Questionnaire part 3 responses

Responses to the seven questions concerning attitudes known to effect patient safety are given in Table 6. Most (71–100%) agreed that they worked in helpful, supportive, and respectful atmosphere; and would speak up if they saw something that might harm a patient. However less than half (43%) felt able to challenge those in authority or to ask questions when they see things that are wrong.

**Table 6:** Responses to statements regarding attitudes known to affect patient safety

	Strongly agree	Agree	Neutral	Disagree	Strongly disagree
We help each other when we are busy	5 (71%)	2 (29%)	0	0	0
When one part of the operating room team is busy, the others all help	5 (71%)	2 (29%)	0	0	0
We treat each other with respect in this operating room	0	5 (71%)	0	1 (14%)	0
We support each other in this operating room	1 (14%)	6 (86%)	0	0	0
People speak up if they see something happening that might harm the patient	2 (29%)	4 (57%)	0	1 (14%)	0
In this hospital we feel free to question those in authority	0	2 (29%)	2 (29%)	1 (14%)	2 (29%)
In this hospital we are afraid to ask questions if we see that things are wrong	1 (14%)	1 (14%)	2 (29%)	3 (43%)	0

#### Observations in the operating room

On the day of the evaluation visit there was no surgery taking place therefore we were unable to observe the SSC in use. However, we were shown the pulse oximeters which showed signs of repeated use, and evidence of counting sheets being used.

### Inhibitors and facilitators to change

Inhibitors to changes in personal and organisational practice were lack of support; and that they had learnt to count in pairs, but were often on their own which made counting difficult. Two facilitators to change were identified: ‘seeing the counting performed made a difference’, and ‘ we all get on well already so we are motivated to help each other’.

## Discussion

In this paper we report that 15 months after a four-day SSC pilot program, the six basic safety processes are being followed by 57–86% of participants; and self-reported positive improvements in teamwork, communication and organisation and safe anaesthesia persist. Evidence of pulse oximeter use and counting of needles, sponges and instruments was also observed.

Our study reports successful SSC implementation after a four-day pilot course in one LMIC hospital. This is a novel finding because to date, successful SSC implementation is reported in just a few single institutions in LMICs and only after sustained time-commitments from HIC providers. [3–7]. Shorter time-scale implementation strategies are needed to facilitate widespread uptake of the SSC in LMICs. Our pilot course offers novel insights in this regard.

The SSC is simple, inexpensive and effective [1] and also has a dose effect [14–16], meaning even if only used in part, surgical outcomes are still improved. Therefore it is clinically significant that in our pilot study the 6 basic safety processes were being followed by 57–86% of participants 15 months after SSC training. Haynes et al. [1] only achieved 67% compliance in all 6 basic safety process measures at 3 months, yet achieved a 47% reduction in mortality, and 50% reduction in post-operative infections. In our study, assessment of the risk of major blood loss, antibiotic prophylaxis given before skin incision, and use of a pulse oximeter (86% of participants) were the most common sustained practice changes at 15 months. It is significant that our participants also experienced positive changes in attitude related to teamwork, organisation and communication, because some studies show that partial completion of the SSC due to poor attitudes can substantially negate the benefits [17].

We specifically designed our pilot programme to take into account our prior lessons learnt [8]; factors known to influence surgical outcomes such as teamwork [18] and the retention of surgical items [19]; and to overcome previously identified implementation barriers such as lack of knowledge, poor buy-in from senior clinicians, inadequate resources and a hierarchical culture [3, 5, 9, 10]. All seven participants reported positive changes in teamwork, organisation and safe anaesthesia, and six out of seven reported improvements in communication. Many participants also noted organisational changes such as accompanying patients from the ward to the operating room, introducing themselves in front of the patient and asking visiting surgeons to do the same. Furthermore there was a perception of high levels of help (100%), respect (71%) and support (86%) among the operating room team. This may partially explain why our short four-day pilot course has sustained success despite the lack of a permanent surgeon/obstetrician. The nurses reported making the temporary surgeons do the SSC and in particular introduce themselves in front of the patient. The high degree of team coherence, culture of respect, as well as supportive hospital director may explain this finding [8, 9, 20].

Key features of course design that differ from previous studies [3–7] and may have enabled a short course 4 day course to succeed include:

1. local ownership
  - involvement of the hospital director from the planning to implementation and follow- up stages
  - time spent understanding the rationale and adapting the SSC to the local environment which was led by the local staff and on going adaptations made through out the course
2. multidisciplinary team training
  - involvement of doctors and nurses working together through out the course and modelling how to speak up and help and support one another
3. operating room simulation
  - allowed the course to be not just theoretical but practical and highlighted challenges not foreseen when using simulation in the classroom

Additionally the hospital was small, and the operating team was a close-knit team as evidenced by one participant who said,

- *We all get on well already so we are motivated to help each other.*

However, other participants commented that as a result of training:

- *We can help each other and encourage each other*
- *We now have a team atmosphere, suggestions and mutual exchanges*
- *Before I would just get angry if people weren’t interested, now we can talk about it*
- *We are nicer to each other*

These statements indicate that the SSC had made a noticeable improvement even if one participant felt the atmosphere was good to start with. Despite most participants (71–100%) reporting a helpful, respectful and supportive atmosphere, one area that still requires more attention is to the breaking down of the hierarchical culture and questioning those in authority. Our results show less than half (43%) of the participants feel able to challenge those in authority or ask questions when they see things that are wrong. This could be because hierarchy and questioning those in authority are implied in the course but not explicitly taught whereas teamwork, communication, organisation and safe anaesthesia are.

Our study has several limitations. The main one is that this is a pilot study at a single, small institution. The data was self-reported and therefore open to subjective bias, both under-reporting out of a desire to gain more training and equipment; or and over-reporting to impress the evaluators or hospital director. We did not measure attitudes to teamwork, communication, organisation and safe anaesthesia; or responses to the statements regarding the safety culture of the hospital, before the study and therefore have no baseline data for comparison. The questionnaire although based on solid Kirkpatrick principles was novel and not previously validated; therefore this was a pilot study in a small hospital and only 7 out of 17 (40%) participants were available for impact evaluation. Therefore results should be interpreted with caution and cultural differences may limit applicability of the course to other countries. The lack of permanent surgeon in this pilot study also limits the general applicability and may have introduced bias. Surgeons can become SSC champions and positively influence implementation, however disengaged surgeons are known to hinder implementation [9, 20]. That participants in this study reported persuading visiting temporary surgeons to introduce themselves and use the SCC shows positive buy-in from nursing staff and a willingness to promote safe surgical practice.

Despite these limitations we believe our study has a number of strengths. It was a pilot study designed to test the hypothesis that sustainable SSC implementation is possible after only a four-day course. Widespread SSC implementation in LMICs requires resource-light novel strategies and despite the small numbers our pilot course adds to the literature in this regard. We used a mix methods analysis based on the Kirkpatrick model of evaluation [12] to evaluate changes in attitudes and determine facilitators and inhibitors to personal and organisational change which will be used in further course development

## Conclusion

Our pilot study shows that in one hospital in the Republic of Congo, SSC implementation was achieved and sustained at 15 months after a four-day pilot training course, with the six basic safety measures being followed by 57–86% of participants. Further work is required to test this model in larger hospitals to assess the suitability of a short course format for wide-scale national implementation.

## Abbreviations

HIC: High Income Country
LMIC: Low Middle Income Country
SSC: Surgical Safety Checklist
WHO: World Health Organisation
